# Effects of Acute Aerobic Exercise on Cognitive Flexibility Required During Task-Switching Paradigm

**DOI:** 10.3389/fnhum.2019.00260

**Published:** 2019-07-31

**Authors:** Seongryu Bae, Hiroaki Masaki

**Affiliations:** ^1^Department of Preventive Gerontology, Center for Gerontology and Social Science, National Center for Geriatrics and Gerontology, Obu, Japan; ^2^Faculty of Sport Sciences, Waseda University, Tokyo, Japan

**Keywords:** acute aerobic exercise, cognitive flexibility, event-related potentials, P3, task-switching

## Abstract

The present study aimed to investigate the effects of acute aerobic exercise on underlying neuronal activities associated with task-switching processes including both mixing and switch effects. A total of 29 healthy young adults (21.4 ± 1.2 years) participated in this study. The experiment consisted of an exercise and a rest condition. In the exercise condition, participants completed 30 min of self-paced motor-driven treadmill exercise at 70% intensity of maximum heart rate (HR_max_). In the rest condition, participants rested quietly for 30 min. Both conditions were conducted on different days, and the order was counterbalanced across participants. Participants performed the task-switching paradigm (switching between two different tasks), after both exercise and rest conditions respectively. The P3 amplitude was smaller in the non-switch trial than the single trial only in the rest condition, but not in the exercise condition. In other words, more attentional resources were allocated to the non-switch trial requiring greater amounts of working memory following the exercise condition. Mixing and switch effects on the P3 component were influenced by acute aerobic exercise. This result suggests that acute aerobic exercise may serve to facilitate the flexibility of task-set reconfiguration and maintain the task set in working memory. Furthermore, during the switch trials, the P3 latency was shorter in the exercise condition than in the rest condition. This result is consistent with the findings of previous studies, indicating that cognitive processing speed is increased only during task conditions requiring greater amounts of executive control. Our study clearly indicates that acute aerobic exercise may facilitate cognitive flexibility as well as other executive functions (i.e., inhibitory control and working memory).

## Introduction

Numerous studies have confirmed the beneficial effects of acute exercise on cognitive functions. Ludyga et al. (2016) suggested that acute aerobic exercise might have maximum benefits for tasks that primarily require cognitive abilities associated with planning, maintaining, and implementing goal-directed behaviors. Contemporary theories pertaining to cognitive neuroscience refer to these cognitive abilities as executive function. The executive function is not uni-dimensional and consists of three components: inhibitory control, working memory, and cognitive flexibility (Miyake et al., 2000).

A growing body of evidence has demonstrated that acute exercise facilitates inhibitory control (e.g., Hillman et al., 2003; Drollette et al., 2014) and working memory (e.g., Coles and Tomporowski, 2008; Pontifex et al., 2009a). However, further research is needed to elucidate the relationship between exercise and cognitive flexibility. Cognitive flexibility represents an ability to adapt to changing environments (Miyake et al., 2000). The task-switching paradigm requires a rapid shifting of cognitive control between two or more tasks (Monsell et al., 2003) and has been widely used to measure cognitive flexibility. It can test both the mixing effect (i.e., difference in performance between mixed-task and single-task blocks) and the switch effect (i.e., difference in performance between switch and non-switch trials within mixed-task blocks; Karbach and Kray, 2009). The mixing and switch effects share different constructs (Kray and Lindenberger, 2000). The mixing effect is thought to reflect the difficulty of maintaining task sets in working memory, whereas the switch effect is thought to reflect the cognitive rigidity or flexibility of task set reconfiguration (Rogers and Monsell, 1995).

A few studies have investigated the effects of acute aerobic exercise on cognitive flexibility, measuring both reaction time (RT) and response accuracy. For example, using a task-switching paradigm, Tomporowski and Ganio (2006) found a shorter RT in the switch trial compared to the non-switch trial during both 40-min aerobic cycling exercise and rest periods, indicating that acute exercise did not facilitate set switching. A follow-up study replicated the null effect of acute exercise on set switching (Coles and Tomporowski, 2008). However, it is still possible that neural activities associated with cognitive flexibility might change after acute exercise. Given that event-related brain potentials (ERPs) associated with other executive functions increased in amplitude (Hillman et al., 2003) and brain-derived neurotrophic factor (BDNF), which is thought to be responsible for the beneficial exercise effect, transiently increased even after a single bout of acute exercise (Winter et al., 2007), it is reasonable to assume that cognitive flexibility may also benefit from acute exercise. Indeed, some studies have reported that behavioral measures and ERPs might represent different effects produced by task manipulations (e.g., Hajcak et al., 2005; Masaki et al., 2017).

The possibility of Type II error due to a relatively small sample (i.e., less than 20 participants) cannot be ruled out in the studies of Tomporowski and Ganio (2006). Furthermore, cognitive function could be influenced by changes in physiological arousal state after acute exercise that is modulated by cortisol levels (Kashihara et al., 2009; Tsai et al., 2014). Thus, we were motivated to clarify underlying neuronal activities during execution of task-switching after a single bout of acute aerobic exercise.

In addition to behavioral measurements, electrophysiological studies recording ERPs have demonstrated the facilitating effect of acute aerobic exercise on cognitive functions throughout the life span. Hillman et al. (2003) confirmed that electrophysiological measures can reveal the underlying mechanisms of acute-exercise benefits more clearly than behavioral measures. ERPs are scalp-recorded voltage deflections, providing information about brain activities associated with stimulus evaluation and response execution with high temporal resolution. Notably, most ERP studies that investigated the effects of acute aerobic exercise on executive control have focused on the P3 component (e.g., Hillman et al., 2003). The P3 is a positive-going deflection emerging approximately 300 ms after the stimulus presentation over centroparietal regions (Polich and Kok, 1995). Its amplitude increases in proportion to the amount of attentional resources allocated to a given task (Donchin and Coles, 1988), and its latency is thought to reflect the stimulus classification speed or stimulus evaluation time (Kutas et al., 1977). Hillman et al. (2003) found a larger P3 amplitude compared to the baseline and a shorter P3 latency for incompatible stimuli than for neutral stimuli in a flanker task following 30-min of acute exercise. A subsequent study also reported similar results (Kamijo et al., 2007). These studies suggest that acute exercise may improve cognitive processing speed during tasks requiring greater amounts of executive control.

Previous studies that examined the effect of acute exercise on executive function reported increased P3 amplitude and decreased P3 latency, suggesting that acute exercise may increase the amount of attentional resources allocated to an executive control task (Hillman et al., 2003). According to these results, it can be assumed that cognitive flexibility (i.e., one of the executive functions) should improve after acute aerobic exercise. The beneficial effects of acute aerobic exercise on performance in a task-switching paradigm by examining P3s have been investigated (Tsai et al., 2016). Tsai et al. (2016) manipulated cardiorespiratory fitness and found a smaller switch cost and larger P3 following acute exercise for the higher cardiorespiratory fitness group. However, they averaged ERPs collapsing different tasks without considering and differences in task difficulty. Because it is well-known that P3 amplitude and latency are affected by task difficulty (e.g., Donchin, 1981; Polich, 1987), we analyzed the P3s separately in each task (i.e., small/large task or odd/even task) and in each switch condition (i.e., switch or non-switch). We were motivated to clarify the beneficial effects of acute exercise on cognitive processing associated with task-switching, considering task difficulty. Furthermore, another difference between our study and Tsai et al.’s (2016) was the predictability of the subsequent task in the mixed task condition. In the current study, two tasks were randomly presented (e.g., ABAABBBA…) in the mixed task condition, whereas in Tsai et al.’s (2016) study, two tasks were alternately presented (i.e., AABBAABB…). The random occurrence of switch trial might have produced a greater amount of interference on the switch trials in association with the prior task set, because the participants could not predict when a switch would occur and thus, they were unable to prepare for new task set. This design may require more cognitive flexibility (Monsell and Mizon, 2006; Friedman et al., 2008). Finally, because Tsai et al.’s (2016) study investigating the effects of acute aerobic exercise have not measured the P3 latency, it remains unclear whether the effect of acute aerobic exercise on P3 latency during the task-switching paradigm.

This study aimed to investigate the effects of acute aerobic exercise on underlying neuronal activities associated with task-switching processes including both mixing and switch effects. Given that executive functions are affected by acute aerobic exercise (Hillman et al., 2003; Kamijo et al., 2007), it is highly possible that both mixing and switch effects would be reduced following acute exercise. In addition, previous studies have reported that acute aerobic exercise selectively improved task performance and increased P3 amplitude during task conditions requiring greater amounts of executive function (Hillman et al., 2003; Kamijo et al., 2007).

In our study, we hypothesized that task demands would be stronger on the switch trials in the mixed task, on the non-switch trials in the mixed task, and in the single task, in this order. Therefore, it is reasonable to predict that after acute exercise, larger P3 amplitudes and shorter P3 latencies should be clearly observable on the switch trials and the non-switch trials in the mixed task compared to the single task.

## Materials and Methods

### Participants

Thirty undergraduate students were recruited from the Faculty of Sport Sciences, Waseda University. None of the participants had any history of neurological diseases or cardiovascular disease. They were right-handed and had normal or corrected to normal vision. One participant was excluded from analyses due to excessive artifacts in the electroencephalogram (EEG) signal. Table 1 summarizes participants’ demographic and fitness information for this sample. Each participant received 1,000 yen/hour (about 10 U.S. dollars) for their participation. This study was approved by the Ethics Committee on Human Research of Waseda University, and written informed consent was provided by all participants prior to the experiment.

**Table 1:** Mean (*SD*) values for participant demographics and fitness data.

	Males (*N* = 14)	Females (*N* = 15)
Age (years)	21.7 (1.2)	21.1 (1.1)
Body mass index (kg/m^2^)	23.1 (1.7)	21.2 (2.0)
VO_2max_ (ml/kg/min)	53.6 (5.9)	40.0 (5.3)
HR_max_ (bpm)	191.4 (7.1)	188.0 (8.8)
RPE_max_	18.7 (0.8)	19.4 (0.5)
HR at exercise condition (bpm)	137.5 (9.2)	131.9 (10.0)
RPE at exercise condition	12.0 (0.8)	12.4 (0.8)
Post-rest HR (bpm)	65.4 (3.8)	68.5 (7.3)
Post-exercise HR (bpm)	66.2 (3.8)	69.0 (7.3)

*All data are present as means (SD). VO_2max_, maximal oxygen consumption; HR_max_, maximal heart rate; RPE_max_, maximal rating of perceived exertion; HR, heart rate; RPE, rating of perceived exertion; Post-rest HR, HR prior to cognitive task during the rest condition; Post-exercise HR, HR prior to the cognitive during exercise condition*.

### Laboratory Procedure

Participants visited the laboratory on three separate days. During the first visit, participants completed the Physical Activity Readiness Questionnaire (PAR-Q; Thomas et al., 1992) and the Edinburgh Handedness Inventory (Oldfield, 1971). To evaluate participants’ cardiorespiratory fitness, the graded exercise test (GXT) was performed on a treadmill. During the second and third visits, participants were assigned to the exercise condition and rest conditions. The order of the exercise and rest condition was counterbalanced across participants. Each condition was conducted at a similar time each day with a mean delay between visits of 4 days in order to minimize potential task practice effects. The exercise condition began with 30 min of self-paced motor-driven treadmill exercise at 70% intensity of maximum heart rate (HR_max_) achieved on the GXT. The cognitive task was executed after participants’ HR returned to within 10% of pre-exercise levels (approximately 20–30 min after acute aerobic exercise). This procedure was based on previous studies (Magnié et al., 2000; Hillman et al., 2003). During the exercise condition, we recorded HR per minute and the rate of perceived exertion (RPE) every 5 min. During the rest condition, participants were seated on a chair and were instructed to read a newspaper for 30 min. Following the 30-min exercise or seated rest, the participant wore an electrode cap for EEG recordings and performed a task-switching task while behavioral data and EEGs were recorded.

### Task-Switching Paradigm

Participants performed a cognitive task that was created according to a modified task-switching paradigm (Allport et al., 1994; Rogers and Monsell, 1995; Hillman et al., 2006). Each trial began with the presentation of a cue stimulus (either solid or dashed square) on the screen for 300 ms that was followed by a target stimulus (200 ms duration). Target stimuli consisted of the digits from 1 to 9, excluding the number 5. The participants were asked to determine whether the subsequent digit was odd (press the left button) or even (press the right button) when the cue appeared in the dashed square, and to decide whether the subsequent digit was smaller (press the left button) or larger (press the right button) than 5 when the cue appeared in the solid square. They were instructed to respond to the target stimuli within 1,000 ms. The target stimuli were randomly presented.

The single block (i.e., the decision of either odd/even or more/less than 5) was composed of 64 trials each. In the odd/even single block, participants only decided whether the target digit was odd or even, whereas in the more/less single block, they only decided the size of the target digit. The mixed block (i.e., task-switching) was composed of 256 trials (64 trials × 4 blocks), including both the non-switch trial and the switch trial. Among the 256 trials in the mixed block, 128 odd/even task (32 trials × 4 blocks) or 128 small/large task (32 trials × 4 blocks) appeared with equal probability in a random order. Furthermore, in the current study, the two tasks were randomly presented (e.g., ABAABBBA…) in the mixed block. This design could produce an additional source of interference from the preceding task-set on the switch trial (Tsai and Wang, 2015). Among the 256 trials in the mixed block, 128 non-switch trials (32 trials × 4 blocks) or 128 switch trials (32 trials × 4 blocks) randomly appeared with equal probability. Task changes occurred after a minimum of one and a maximum of nine intervening trials. The first trial in each block was excluded from the analyses. The behavioral and ERP data were analyzed separately: (1) single block (i.e., odd/even and small/large task); (2) non-switch trial in the mixed block (i.e., repeated the same task as required on the preceding trial); and (3) switch trial in the mixed block (i.e., switched the task from the type required on the preceding trial). Prior to beginning each task block, they practiced using a block of 64 practice trials. The order of blocks was counterbalanced among the participants.

### ERP Recording

EEG was recorded from 128 electrode sites on the scalp with the Biosemi Active-Two system (Biosemi Inc., Netherland). Vertical and horizontal electrooculograms (EOG) were also simultaneously recorded from electrodes placed above and below the left eye and placed on the outer canthus of each eye, respectively. These signals were recorded with DC to 205 Hz and digitized at a rate of 1,024 Hz. Using the BrainVision Analyzer (Brain Products), EEG data were re-referenced with average reference and passed through a bandpass filter from 0.1 Hz to 30 Hz. The EOG artifacts were corrected using an algorithm described by Gratton et al. (1983). Trials including response error and EEG drifts exceeding ±100 μV were excluded from the analyses. The EEG data were segmented from −100 ms to +800 ms relative to the target stimulus onset and averaged. Baseline was corrected using mean voltage of the 100 ms pre-target window. Because P3 exhibits maximum amplitudes over parietal regions (Polich and Kok, 1995), we scored mean voltages at Pz as the P3 amplitude using a time window of 280–380 ms following the target stimulus onset. The P3 latency was scored as the interval between the target stimulus onset and the positive peak of P3.

### Cardiorespiratory Fitness Assessment

GXT was performed according to a treadmill ramp protocol, which was designed to increase the speed and grade of metabolic equivalents (MET) per minute. A computerized indirect calorimetry system collected breath-by-breath values for oxygen consumption and respiratory exchange ratio (RER). The HR was recorded throughout the test, and the RPE was assessed per minute (Borg, 1970). The GXT was terminated when the participants achieved at least two of the three following criteria: (1) a peak HR at or above 95% of age-predicted HR_max_; (2) RPE > 18; and (3) RER > 1.10 (Davis et al., 1997; Santos and Giannella-Neto, 2004).

### Statistical Analysis

To analyze mixing and switch effects, three-way mixed-model analysis of variance (ANOVA) was conducted on behavioral data, P3 amplitude, and P3 latency. Mixing effect was analyzed by including the following factors: condition (resting vs. exercise) × Task (small/large vs. odd/even) × Trial (single vs. non-switch). Switch effect was analyzed by including the following factors: condition × Task × Trial (non-switch vs. switch). Greenhouse-Geisser corrections were applied when the assumption of sphericity was violated. To test interactions, *post hoc* tests were conducted using the Bonferroni correction. The significance level was set at 0.05.

## Results

### Performance

#### Mixing Effects

**Figure 1:**
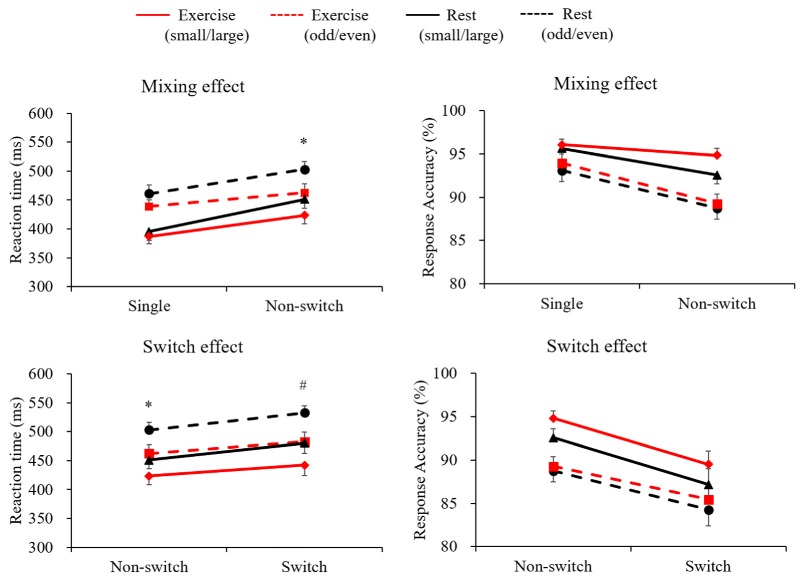
Mean reaction time (RT) and response accuracy across conditions and tasks for the mixing effect (single trial vs. non-switch trial: upper panels) and the switch effect (non-switch trial vs. switch trial: lower panels). Error bars indicate standard error of mean. *The exercise condition showed a significantly shorter RT than the rest condition in the non-switch trial. ^#^The exercise condition showed a significantly shorter RT than the rest condition in the switch trial.

Figure 1 illustrates both RT and response accuracy for each trial and condition. The RT following the exercise condition was shorter than the in the rest condition (*F*_(1,28)_ = 10.89, *p* = 0.003, $\eta_{\text{p}}^{2}$ = 0.28). A marginally significant Condition × Trial interaction (*F*_(1,28)_ = 3.78, *p* = 0.062, $\eta_{\text{p}}^{2}$ = 0.12) was found, with *post hoc* tests indicating shorter RTs in the exercise condition than in the rest condition only for the non-switch trials (*p* = 0.002, *d* = 0.432) and not for the single trials (*p* = 0.065, *d* = 0.209). Response accuracy was higher for the single trials than for the non-switch trials (*F*_(1,28)_ = 22.1, *p* < 0.001, $\eta_{\text{p}}^{2}$ = 0.44), and was higher in the small/large task than in the odd/even task (*F*_(1,28)_ = 25.9, *p* < 0.001, $\eta_{\text{p}}^{2}$ = 0.48). However, neither a main effect nor an interaction involving Condition factor was significant.

#### Switch Effects

A three-way ANOVA on mean RTs in terms of switch effect revealed significant main effects of Condition (*F*_(1,28)_ = 14.04, *p* = 0.001, $\eta_{\text{p}}^{2}$ = 0.33), Task (*F*_(1,28)_ = 96.26, *p* < 0.001, $\eta_{\text{p}}^{2}$ = 0.77), and Trial (*F*_(1,28)_ = 41.25, *p* < 0.001, $\eta_{\text{p}}^{2}$ = 0.59), indicating that the RTs were shorter in the exercise condition than in the rest condition, shorter in the small/large task than in the odd/even task, and shorter in the non-switch trials than in the switch trials. A significant Condition × Trial interaction (*F*_(1,28)_ = 4.98, *p* = 0.034, $\eta_{\text{p}}^{2}$ = 0.15) was also found. *Post hoc* tests indicated shorter RTs in the exercise condition than in the rest condition both for the non-switch (*p* = 0.002, *d* = 0.432) and the switch trials (*p* = 0.001, *d* = 0.505). Response accuracy was higher for the non-switch trials than for the switch trials (*F*_(1,28)_ = 38.0, *p* < 0.001, $\eta_{\text{p}}^{2}$ = 0.58), and was higher in the small/large task than in the odd/even task (*F*_(1,28)_ = 18.2, *p* < 0.001, $\eta_{\text{p}}^{2}$ = 0.39). However, no main effects nor an interaction involving the Condition factor was significant.

### P3 Component

#### Mixing Effects

**Figure 2:**
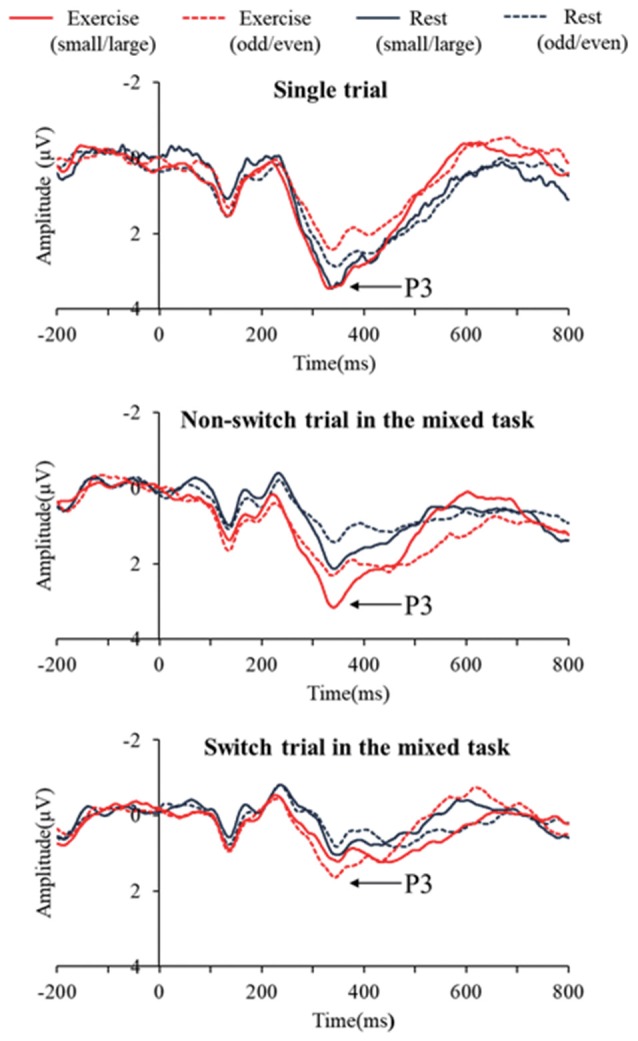
Grand averaged event-related brain potential (ERP) waveforms at Pz across conditions and tasks for each trial.

**Figure 3:**
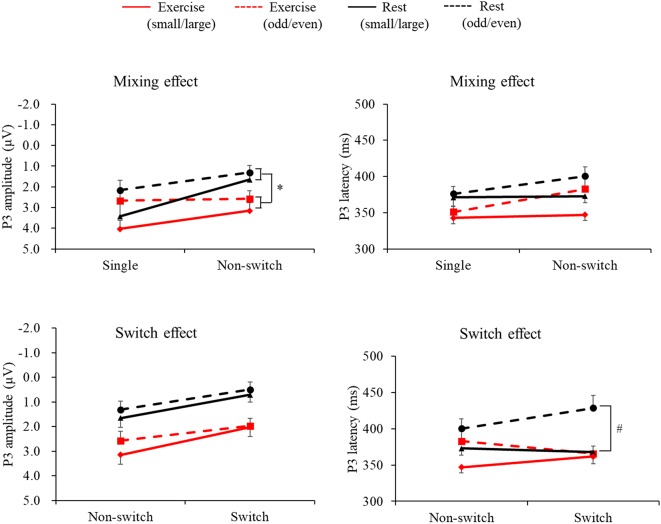
Mean P3 latencies and amplitudes across conditions and tasks for the mixing effect (single trial vs. non-switch trial: upper panels) and the switch effect (non-switch trial vs. switch trial: lower panels). Error bars indicate standard error. *The exercise condition had a significantly larger P3 amplitude than the rest condition in the non-switch trial (*p* = 0.001). ^#^P3 latency was shorter in the exercise condition than in the rest condition only for the switch trials (*p* = 0.002).

Figure 2 presents the grand averaged ERP waveforms for each condition and trial at the Pz electrode site. Figure 3 presents mean P3 amplitudes and latencies associated with the mixing and switch effects across conditions and tasks for each trial. The P3 amplitude was larger in the exercise condition than in the rest condition (*F*_(1,28)_ = 7.51, *p* = 0.01, $\eta_{\text{p}}^{2}$ = 0.21), larger in the small/large task than the odd/even task (*F*_(1,28)_ = 14.92, *p* = 0.001, $\eta_{\text{p}}^{2}$ = 0.35), and larger for the single trial than the non-switch trial (*F*_(1,28)_ = 10.70, *p* = 0.003, $\eta_{\text{p}}^{2}$ = 0.28). In addition, an interaction of Condition by Trial was also significant (*F*_(1,28)_ = 5.11, *p* = 0.032, $\eta_{\text{p}}^{2}$ = 0.15). *Post hoc* tests revealed that increased P3 in the exercise condition relative to the rest condition was observed only in the non-switch trials (*p* = 0.001, *d* = −0.683) and not in the single trials (*p* = 0.20, *d* = −0.182).

The P3 latency was shorter in the exercise condition than in the rest condition (*F*_(1,28)_ = 12.41, *p* = 0.001, $\eta_{\text{p}}^{2}$ = 0.31). In addition, the P3 latency was shorter in the small/large task than the odd/even task (*F*_(1,28)_ = 5.70, *p* = 0.024, $\eta_{\text{p}}^{2}$ = 0.17) and shorter in the single trials than the non-switch trials (*F*_(1,28)_ = 7.43, *p* = 0.011, $\eta_{\text{p}}^{2}$ = 0.21).

#### Switch Effects

A three-way ANOVA on the P3 amplitude revealed that P3 amplitudes were larger in the exercise condition than the rest condition (*F*_(1,28)_ = 17.78, *p* < 0.001, $\eta_{\text{p}}^{2}$ = 0.38), larger for the small/large task than the odd/even task (*F*_(1,28)_ = 6.41, *p* = 0.017, $\eta_{\text{p}}^{2}$ = 0.18), and larger for the non-switch trial than the switch trial (*F*_(1,28)_ = 30.48, *p* < 0.001, $\eta_{\text{p}}^{2}$ = 0.52). No interaction was found (*F*’s < 1.45).

Another three-way ANOVA on the P3 latency revealed a Condition × Task × Trial interaction (*F*_(1,28)_ = 7.93, *p* = 0.009, $\eta_{\text{p}}^{2}$ = 0.22). A simple effects analysis examining Condition × Trial for each task revealed a significant Condition × Trial interaction effect in the odd/even task (*F*_(1,28)_ = 4.43, *p* = 0.04, $\eta_{\text{p}}^{2}$ = 0.14). *Post hoc* tests showed that P3 latency was shorter in the exercise condition than in the rest condition only for the switch trials (*p* = 0.002, *d* = 0.926). No interaction was found in the small/large task.

## Discussion

The present study examined changes in cognitive flexibility associated with task-switching after acute exercise, recording ERPs as well as behavioral measurements. For both mixing and switch effects, we obtained main effects of condition, showing shorter RTs, larger P3 amplitudes, and shorter P3 latencies in the exercise condition compared to the rest condition. Focusing on the mixing effect, however, the increased P3 in the exercise condition was found only for the non-switch trials and not for the single trials. For the switch effect, the shorter P3 latency in the exercise condition was found only for the switch trials and not for the non-switch trials. The acute exercise effect appeared to rely on the task difficulty (i.e., higher task demanding) and the trial type according to the obtained interactions.

For the mixing effect, we found enhanced P3 following exercise on the non-switch trials in the switch task. An increase in P3 amplitude is thought to reflect an increase in mental workload (Donchin, 1981) and in the amount of attentional resources allocated to a given task (Polich, 1987; Kramer and Strayer, 1988). In the task-switching paradigm, the mixing effect reflects the ability to maintain task sets in the working memory, responding to loading multiple tasks (Kray and Lindenberger, 2000). Previous studies have reported decreased P3s in the non-switch trials relative to the single trials, reflecting a small amount of available attentional resources (Goffaux et al., 2006; Jost et al., 2008) due to higher task demands in the non-switch trials. Our results are consistent with previous findings. For the exercise effect, the enhanced P3 found in the non-switch trials following exercise might also be due to better allocation of available attentional resources. It is highly possible that the beneficial effects of exercise tend to emerge in a higher task-demanding situation (i.e., non-switch trials), but not in an easy task (i.e., single trials).

The P3 latency was shorter in the single trials than in the non-switch trials regardless of exercise. There was no significant interaction between condition (exercise/rest) and trial (single/non-switch), suggesting the similar temporal processing of the perceptual/central stage between conditions (Kok, 1997; Polich, 1997). Because the pre-cue provided task-relevant information prior to the target stimulus, the absence of difference in P3 latency as a mixing effect might be due to lower working memory demands.

For the switch effect, we reconfirmed longer RTs in the switch trials than in the non-switch trials. In addition, RT was shorter following acute exercise than following rest on the switch trials, but not on the non-switch trials. The shortened P3 latency as a result of acute aerobic exercise was found only for the switch trials in the odd/even task. As observed in the mixing effect, the exercise effect tended to emerge in a higher workload situation even for the switch effect. According to longer RTs, longer P3 latencies, and lower response accuracies in the odd/even task, the switch trials in the odd/even task appeared to be the most difficult to perform. The reconfiguration of a task set is believed to rely on resource demanding control that results in longer RTs on switch trials than on non-switch trials (Coles and Tomporowski, 2008). Our results suggest that acute aerobic exercise may facilitate stimulus evaluation and classification on the switch trials.

The task was randomly changed between two tasks (from small/large to odd/even, vice versa) in our study. The random occurrence of switch trial might have produced a greater amount of interference from the prior task set on the switch trials, because the participants could not predict when a switch would occur and were thus unable to prepare for a new task set. This design may require more cognitive flexibility (Monsell and Mizon, 2006; Friedman et al., 2008). It is plausible that the amount of executive control involved in the cognitive task influences the relationship between acute exercise and cognitive flexibility because beneficial exercise effects emerged only when the switch task involved the effortful maintenance of a high amount of executive control. Although there was no significant interaction between condition (exercise/rest) and trial (non-switch/switch) for the P3 amplitude due to the switch effect, the results showed larger P3 amplitudes in the exercise condition compared to the rest condition in the task-switching paradigm. This suggests that the beneficial effect of acute aerobic exercise on task-switching may be associated with improved attentional resource allocation.

In the present study, no significant effect of acute aerobic exercise on response accuracy was found. Previous studies testing middle-aged adults found that acute exercise resulted in higher response accuracy in a Stroop test and the Tower of London task (Chang et al., 2012, 2014). On the other hand, and in accordance with our study, other studies testing young adults did not find any effect of acute aerobic exercise on response accuracy in Ericksen flanker tasks and task-switching task (Hillman et al., 2003; Tsai et al., 2016). Although the response accuracy did not differ between the rest and exercise conditions in our study, it deteriorated with increasing task difficulty, suggesting success in the task-switching manipulation. A possible reason for the null result in our study might be due to a ceiling effect in young adults.

The timing of the cognitive assessment following acute aerobic exercise should be also considered because body temperature and HR positively correlate with P3 amplitudes (Geisler and Polich, 1990; Tsai et al., 2016). Hillman et al. (2003) found that P3 increased in amplitude across conditions in a flanker task approximately 48 min following moderately high-intensity exercise when participants’ HR had already returned to baseline level. Tsai et al. (2016) showed a smaller switching cost and larger P3 amplitude 15–20 min following 30-min of acute aerobic exercise when HR and temperature had returned to within 10% of pre-exercise levels. Magnié et al. (2000) revealed larger P3 amplitude and shorter latency after a GXT, even when the data was collected immediately after temperature and HR had returned to pre-exercise values. These studies have conducted neuroelectric testing 15–48 min following acute exercise. Our results are consistent with the results of these findings, suggesting that the effect of acute aerobic exercise on P3 might be sustained even after the restoration of HR to pre-exercise level (on average about 20–30 min after acute aerobic exercise).

The underlying neuronal mechanisms of the acute exercise effect have been explained by a transient augmentation of neurochemicals as well as increases in regional cerebral blood flow (CBF) following acute aerobic exercise (e.g., Querido and Sheel, 2007; Pontifex et al., 2009b). Considering that acquisition of exercise habits is a result of the repetition of acute exercise, both types of exercise effects might rely on similar neuronal activities. Acute exercise may increase a variety of neurochemicals such as lactate, cortisol, neurotrophins, neurotransmitters, and neuromodulators, which would result in beneficial acute exercise effects (for a systematic review, see Basso and Suzuki, 2017). Particularly, cortisol modulates arousal (Lambourne and Tomporowski, 2010), and acute aerobic exercise can also increase the arousal state and neural activation (Magnié et al., 2000). Given the conceptual link between cortisol and exercise, cortisol might be responsible for the effects of exercise on cognitive performance (Henckens et al., 2012). Previous studies have suggested that the relationship between acute aerobic exercise and cognitive performance may be mediated by changes in peripheral BDNF concentrations (Vaynman and Gomez-Pinilla, 2005; Winter et al., 2007). Although it remains unclear if peripheral BDNF level can mirror central BDNF level, these findings suggest that a transient increase of BDNF following a single bout of intense acute exercise might be responsible for the beneficial effects on cognitive functions. Even though we tested lower intensity exercise (70% HR_max_) relative to these studies (ventilatory threshold + 10% and 80% HR_max_), it was likely sufficient to increase central BDNF level (e.g., Ferris et al., 2007; Schmolesky et al., 2013).

On the other hand, there is also a possibility that the underlying mechanisms may differ between chronic and acute exercise effects. Thus, it is suggested that each exercise effect on executive functions should be examined separately. Gutmann et al. (2015) emphasized that increased CBF following acute exercise may activate arousal mechanisms and result in efficient information processing. Therefore, increased arousal level due to acute aerobic exercise might be another potential mechanism of the acute exercise effect, facilitating the release of brain neurotransmitters such as catecholamines and acetylcholine and, thereby, physiological readiness to respond as well as peripheral changes in HR (Hillman et al., 2003; McMorris and Hale, 2012; Byun et al., 2014; Thacker et al., 2014; Chang et al., 2015). In addition, it is plausible that arousal level did not differ among trials but differed between the exercise and rest conditions in our study.

It should be also noted that a previous study found no relationship between cardiovascular fitness and the P3 component in the context of cognitive flexibility (i.e., no chronic exercise effect; Scisco et al., 2008). Because they did not test the effects of acute exercise on cognitive flexibility, it is unclear whether or not the discrepancy between their null result and our findings can be ascribed to chronic and acute exercise effects, respectively. Further study is needed to clarify if chronic exercise has no impact on cognitive flexibility.

Lastly, we should refer to a limitation in our study. We did not assess lifestyle habits, including medical history, smoking habits, exercise habits, general cognitive function, and depressive symptoms, which may be important covariates in the relationship between exercise and cognitive functions. Thus, these covariates should be also considered in future studies.

## Conclusion

The findings of this study indicate that acute aerobic exercise may facilitate flexibility of task-set reconfiguration and efficiency in maintaining task set in working memory. In addition, it suggests that task demand is a critical factor to obtain acute exercise effect. These findings may provide a basis for task setting when the task difficulty is properly determined in a study that examines the exercise effect on cognitive functions.

## Ethics Statement

This study was approved by the Ethics Committee on Human Research of Waseda University, and written informed consent was provided by all participants prior to the experiment.

## Author Contributions

SB and HM designed and performed the experiments, analyzed and interpreted the data, and wrote the article and contributed directly to the work and approved it for publication.

## Conflict of Interest Statement

The authors declare that the research was conducted in the absence of any commercial or financial relationships that could be construed as a potential conflict of interest.
